# Replication protein A protects lagging strand gaps, restricting PARP inhibitor-induced synthetic lethality in BRCA1-deficient tumors

**DOI:** 10.1093/nar/gkag396

**Published:** 2026-04-28

**Authors:** Pamela S VanderVere-Carozza, Matthew R Jordan, Joy E Garrett, Karen E Pollok, Katherine S Pawelczak, John J Turchi

**Affiliations:** Department of Biochemistry, Molecular Biology and Pharmacology, Indiana University School of Medicine, Indianapolis, IN 46202, United States; Department of Biochemistry, Molecular Biology and Pharmacology, Indiana University School of Medicine, Indianapolis, IN 46202, United States; Indiana University Cancer Center, Indiana University School of Medicine, Indianapolis, IN 46202, United States; Department of Radiation Oncology, Indiana University School of Medicine, Indianapolis, IN 46202, United States; Herman B. Wells Center for Pediatric Research, Departments of Pediatrics, Biochemistry, Molecular Biology and Pharmacology, Medical and Molecular Genetics, Indiana University, Indianapolis, IN 46202, United States; Indiana University Cancer Center, Indiana University School of Medicine, Indianapolis, IN 46202, United States; NERx BioSciences, Indianapolis, IN 46220, United States; Department of Biochemistry, Molecular Biology and Pharmacology, Indiana University School of Medicine, Indianapolis, IN 46202, United States; Indiana University Cancer Center, Indiana University School of Medicine, Indianapolis, IN 46202, United States; NERx BioSciences, Indianapolis, IN 46220, United States

## Abstract

Replication Protein A (RPA) is a key single-stranded DNA (ssDNA)-binding protein essential for maintaining genome integrity during DNA replication, repair, and recombination. In this study, we elucidate the mechanisms by which a small-molecule RPA inhibitor induces functional RPA exhaustion. Using non-small cell lung cancer and BRCA1-deficient breast and ovarian cancer models, we demonstrate that RPA is critical for sustaining replication fork speed under normal conditions and for facilitating replication restart following fork stalling. Disruption of replication fork-associated processes, including Okazaki fragment processing and ssDNA gap suppression, increases cellular dependence on RPA for ssDNA protection. Chemical inhibition of RPA exacerbates genome instability in BRCA1-deficient cancer models treated with PARP inhibitors, leading to loss of ssDNA gap protection, chromosome shattering, and ultimately, cell death. Combining genetic and pharmacologic approaches to induce ssDNA accumulation alongside RPA exhaustion *in vivo* shows therapeutic efficacy in BRCA1-deficient breast cancer. These findings provide a mechanistic framework for targeting RPA-mediated ssDNA protection as a therapeutic strategy in cancers experiencing endogenous or therapy-induced replication stress.

## Graphical Abstract

**Graphical Abstract:**
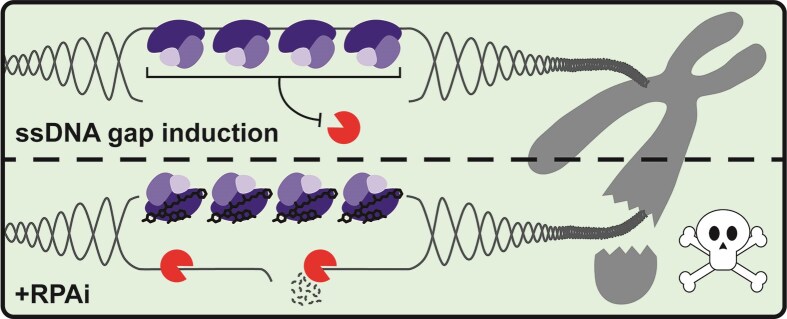

## Introduction

Recent advances in our understanding of DNA damage response (DDR) signaling have opened up an array of opportunities to treat human cancers [1]. The DDR is initiated by phosphatidylinositol 3-kinase-related kinases (PIKKs) ATM, ATR, and DNA-PK, each of which responds to different DNA structures [2]. Each PIKK requires a sensor protein to bind to DNA: Ku binds to double-stranded DNA breaks (DSBs) to activate DNA-PK, MRN binds to DSBs to activate ATM, and replication protein A (RPA) binds to single-stranded DNA (ssDNA) to activate ATR. Once activated, these kinases signal to downstream kinases and other effector proteins to regulate the cell cycle and DNA replication and promote DNA repair. If the damage is not repaired, cell death occurs via apoptosis or mitotic catastrophe. RPA, a human ssDNA-binding protein, is a crucial sensor of elevated ssDNA levels resulting from replication stress (RS) and DNA damage [3]. Binding of the ATR-interacting protein (ATRIP) to the RPA–ssDNA complex results in the activation of ATR and initiation of the DDR [4], limiting replication origin firing and inducing cell cycle arrest to maintain genome stability. The importance of RPA in the protection and signaling events associated with RS has led to a model establishing an RPA protection threshold where sufficient RPA is necessary to protect the ssDNA in a cell [3, 5]. RS induced by oncogene activation results in increased ssDNA via dysregulated helicase loading, premature origin firing, and increased replication initiation [6]. RS induced by DNA damage, replication fork stalling, or chemical inhibition of the DDR can also elevate the ssDNA level above the RPA protection threshold leading to cell death [5, 6].

Considering the central role of RPA in the DDR and RS response, we developed a small-molecule RPA inhibitor (RPAi), NERx-329, which blocks the interaction between RPA and ssDNA [7]. RPAi treatment has broad single-agent anticancer activity and enhances the activity of DNA-damaging therapeutics and DDR targeted agents in combination treatment regimens [8, 9]. Despite excellent synergy with cisplatin, RPAi treatment does not alter nucleotide excision repair (NER)-catalysed removal of cisplatin-DNA lesions [9]. While, RPAi exhibit potent inhibition of DDR signalling *in vitro* [10], cellular combinations with DDR-targeted agents failed to reveal an ATR-dependent mechanism of cytotoxicity [9]. These data led us to assess the importance of RPA protection of ssDNA, particularly in the RS response.

PARPi therapy has been approved for treating a variety of cancers that harbor genetic defects in homologous recombination (HR) via synthetic lethal interactions [11]. More recently, ssDNA gaps have been implicated in the sensitivity of BRCA1-deficient cells to PARP inhibitors [12, 13]. Beyond DSB repair, BRCA1 deficiency results in the formation of lagging strand gaps that can be exacerbated by PARP inhibition, indicating that lagging strand ssDNA gaps are an important determinant of PARPi sensitivity [14, 15]. In this context, ssDNA gaps generated in the initial S phase persist through the cell cycle and become one-ended DSBs during replication in the next S phase, thereby resulting in replication fork collapse [16, 17]. Moreover, in BRCA1-deficient cells with acquired PARPi resistance, excessive ssDNA gaps can still be generated through nuclease-dependent expansion independent of PARPi treatment [18, 19].

In this study, we elucidate the mechanistic impact of RPA exhaustion in the context of unperturbed replication and genetic and pharmacologically induced RS. We demonstrate RPA is critical for sustaining replication fork speed under normal conditions and for facilitating replication restart following fork stalling. Chemical inhibition of RPA exacerbates genome instability, leading to chromosome shattering, and ultimately, cell death and can be exploited *in vivo* as an anticancer therapeutic strategy.

## Materials and methods

### Cell culture

A549 non-small cell lung cancer (NSCLC) cells were cultured in F12-K (Corning) and MDA-MB-436 cultured in 1:1 DMEM:Ham’s F12 medium (Corning), both supplemented with 10% FBS and penicillin/streptomycin. UWB1.298 cells were grown in 1:1 MEGM (Lonza):RPMI (Corning) supplemented with 3% FBS. Media for the UWB1.289 BRCA1 complemented cells included 200 µg/ml G-418 (Sigma). All the cell lines were cultured in 5% CO_2_ at 37°C, and CCK-8 viability assays were conducted as previously described [9].

### DNA fiber combing

#### Replication fork speed

A549 or MDA-MB-436 cells(4 × 10^5^) were plated in a six-well plate and grown for 24 h at 37°C with 5% CO_2_. Cells were treated with vehicle control or 30 µM NERx-329 for 2 h before labeling active replication forks with IdU (50 μM) for 30 min. Cultures were briefly washed three times with pre-warmed phosphate-buffered saline (PBS) and CldU (250 μM) was added for 30 min. The cells were then washed three times with pre-warmed PBS, trypsinized, and embedded in agarose plugs. Agarose plugs were lysed overnight with 2 mg/ml proteinase K (Thermo Fisher Scientific) at 50°C. The next day, the agarose plugs were washed three times with TE buffer for 1 h at room temperature with gentle rotation. Agarose plugs were then added to 0.5 M MES (pH 5.5) at 68°C for 20 min, followed by incubation at 42°C for 10 min. β-agarase (New England Biolabs) was added to digest the agarose and release the DNA overnight at 42°C. The next day, the released DNA mixture was mixed 1:1 with 0.5 M MES (pH 5.5) before the DNA was stretched onto coverslips with a FiberComb machine (Genomic Vision). The coverslips were then incubated at 60°C for 2 h. DNA was denatured by incubating coverslips in 0.5 M NaOH/1 M NaCl. The coverslips were washed three times with PBS, dehydrated with an ethanol series (70%, 90%, and 100%), and air-dried for 30 min. The coverslips were blocked with 3% BSA in PBST (PBS + 0.1% Tween 20) for 1 h at 37°C. The coverslips were incubated with 1/25 mouse anti-BrdU (BD Biosciences, 347580, for IdU detection) and/or 1/50 rat anti-BrdU (Abcam, Ab6326, for CldU detection) in 3% BSA in PBST for 1 h at 37°C. Coverslips were washed three times with PBST and incubated with 1/100 goat anti-mouse cross-adsorbed AlexaFluor488 (Invitrogen) and/or 1/100 goat anti-rat cross-adsorbed AlexaFluor594 (Invitrogen) in 3% BSA in PBST for 1 h at 37°C. The coverslips were washed three times with PBST, mounted on slides with ProLong Diamond antifade mounting medium, and allowed to cure overnight. DNA fibers were visualized using an EVOS FL Auto 2 Imaging System (Invitrogen) under a 60× oil immersion lens with GFP and Texas Red filters. Individual fibers were measured using ImageJ software. All fiber combing data were obtained from duplicate experiments, with a minimum of 100 fibers analyzed for each condition.

#### Replication restart

A549 or MDA-MB-436 cells were (4 × 10^5^) plated in a six-well plate and grown for 24 h at 37°C with 5% CO_2_. The cells were pulsed with 50 µM IdU for 30 min, briefly washed three times with pre-warmed PBS, and treated as indicated with DMSO, 4 mM hydroxyurea (HU), and/or 50 µM mirin for 30 min. The final concentration of DMSO used was 0.1%. The cells were then washed three times with pre-warmed PBS and treated for 1 h with 250 µM CldU, DMSO, and/or 30 µM NERx-329. The final DMSO concentration was 0.6%. Cells and coverslips were processed as described for replication fork speed experiments.

#### Gap suppression

MDA-MB-436 cells (5 × 10^5^) were plated in a six-well plate and grown for 24 h at 37°C with 5% CO_2_. The cells were then treated with DMSO, 10 µM olaparib, 50 µM mirin, and/or 30 µM NERx-329 for 2 h. The final concentration of DMSO was 0.7% for all treatment combinations. After 2 h, the medium was aspirated, and fresh medium was added as indicated with DMSO, 250 µM CldU, 50 µM mirin, and/or 30 µM NERx-329 for 1 h. The cells were then washed thrice with pre-warmed PBS and trypsinized. The cells from each condition were then evenly split into two agarose plugs (for eventual ± S1 nuclease treatment) and lysed overnight with 2 mg/ml proteinase K (Thermo Fisher Scientific) at 50°C. The next day, the agarose plugs were washed three times with TE buffer for 1 h at room temperature with gentle rotation. Agarose plugs were then added to 50 mM MES (pH 5.5) and 100 mM NaCl and incubated at 68°C for 20 min, followed by incubation at 42°C for 10 min. β-agarase (New England Biolabs) was added to digest the agarose and release the DNA overnight at 42°C. The following day, the released DNA mixture was mixed 1:1 with 60 mM sodium acetate (pH 4.6), 20 mM Zn acetate, 100 mM NaCl, and 10% glycerol ± 40 U/mL S1 nuclease (Thermo Fisher). The mixture was incubated at room temperature for 30 min before DNA was stretched onto coverslips using a FiberComb machine (Genomic Vision). Coverslips were processed as described for replication fork speed experiments.

### Neutral comet assay

MDA-MB-436 cells (7.5 × 10^4^) were plated in a 12-well plate and grown for 24 h at 37°C with 5% CO_2_. After 24 h, the cells were treated with DMSO control, 10 µM olaparib, 30 µM NERx-329, or the combination at a final concentration of 0.7% DMSO for 2 h. After 2 h, the medium was removed, the cells were washed twice with PBS, and trypsinized. Cells were diluted to 2 × 10^5^ cells/ml in PBS and mixed (5 μl) with pre-warmed Comet LMAgarose (50 μl; Bio-Techne). The cell-agarose mixture was immediately added onto CometSlides (Bio-Techne) and allowed to solidify. Slides were submerged in lysis buffer (10 mM Tris, pH 8, 2.5 M NaCl, 0.1 mM EDTA, 1% N-laurylsarcosiine, 1% Triton X-100, 1% DMSO) for 1.5 h at 4°C. Slides were washed three times in TBE (pH 8.5) and electrophoresed at 21 V for 30 min. Slides were rinsed three times with cold water, fixed with 70% EtOH at -20°C for 10 min, then dried overnight. The following day, agarose pads were incubated with SYBR green for 30 min, washed once with water, and air-dried before being visualized using an EVOS FL Auto 2 Imaging System (Invitrogen) under 10× magnification with GFP filters. Fifty comets were scored using OpenComet for each replicate.

### CRISPR Screen

A549 cells stably expressing Cas9 (Geneocopia) were plated at 5 × 10^3^ cells/well in 96-well plates and incubated at 37°C, 5% CO_2_ for 24 h. Cells were transfected with 0.2 μl/well Dharmafect1 in Opti-MEM (Gibco) with Edit-R crRNA:tracrRNA complexes (Horizon) at a final concentration of 25 nM. Two hundred twenty-nine individual genes were screened, using the Edit-R synthetic sgRNA library (Horizon GC-006005, Lot 18112). Each gene had four different crRNAs directed against it and each of those four were tested in triplicate. Twenty-four hours after transfection cells were treated with 2uM 329 and incubated an additional 48 h. Viability was assessed by CCK-8 assay (Dojindo). Individual guides that resulted in a 20% reduction in viability were deemed positive. Individual genes were ranked based and the number of positive hits in the 12 replicates for each gene.

### Metaphase spreads

MDA-MB-436 cells (2 × 10^5^) were plated in a 6-well plate and grown for 24 h at 37°C with 5% CO_2_. After 24 h, the cells were treated with 2 µM olaparib or the DMSO control for 2 days at 37°C with 5% CO_2_. Either 5 or 10 µM NERx-329 or DMSO control was added for one additional day at 37°C with 5% CO_2_. The cells were then treated with colcemid for 3 h at 37°C and 5% CO_2_. The medium was removed, the cells were washed twice with PBS, and trypsinized. All media and washes were combined with trypsinized cells and centrifuged. The cells were incubated in 0.56% KCl (w/v) for 15 min at room temperature. The cell suspension was centrifuged, and the cells were fixed in methanol: acetic acid (3:1) on ice for 1 h. The fixed cells were centrifuged and resuspended in a small volume of fixative solution and then dropped onto alcohol-cleaned slides at a height of 6–12 in. The slides were allowed to dry at room temperature for 30 min, and then at 42°C for 10 min. Coverslips were mounted using the Vectashield antifade mounting medium containing DAPI. Chromosomes were visualized using an EVOS FL Auto 2 Imaging System (Invitrogen) with a 60× oil immersion lens and DAPI filter. For each replicate experiment, 50 chromosomes were counted for each experimental condition.

### Micronuclei assessment and quantification

MDA-MB-436 cells were plated in 8-well chamber slides (CellTreat, #229168) at 4 × 10^4^ cells per chamber and allowed to adhere overnight. The cells were treated with RPAi NERx-329, PARPi, or drug combinations for 2 h, as indicated. The amount of DMSO used was kept constant at 0.5% for all the treatments. After 48 h of treatment, 3.5 µg/ml cytochalasin-B in 0.5% DMSO was added to each well to induce cell cycle arrest. After 24 h of cytochalasin-B exposure, the media and chambers were removed, and the cells were incubated with a solution of 45% PBS, 45% 0.075 µM KCl, and 10% 25:1 methanol:acetic acid for 10 min, followed by another 10 min incubation with only the 25:1 methanol: acetic acid mixture. The slide was then washed three times with PBS, and a coverslip was mounted using Vectashield H-1500 with DAPI and allowed to cure at room temperature for 15 min before being stored overnight at 4°C in the dark. Images were captured using an EVOS FL Auto2 microscope, and an average of 100 cells were scored manually for the presence of micronuclei (MN).

### Flow cytometry

MDA-MB-436 cells were plated in 12-well dishes at a density of 2 × 10^5^ cells/well and allowed to adhere overnight. The cells were treated with RPAi NERx-329, the indicated PARPi, or drug combinations for 48 h, as indicated. The DMSO concentration was maintained at 0.5% for all treatments. To identify actively replicating cells, cultures were labeled with EdU for the final 30 min of the 48-h treatment period. After treatment, cells were washed twice with PBS, trypsinized, and collected in PBS containing 1% FBS and 1 mM EDTA. After centrifugation at 400 × *g* for 3 min, the cells were washed once with 1% BSA in PBS. EdU was detected using the Click-iT^®^ EdU Flow Cytometry Assay Kit (Molecular Probes), according to the manufacturer’s protocol. Briefly, the cells were fixed in Click-iT^®^ fixative for 15 min at room temperature and permeabilized with Click-iT^®^ saponin-based permeabilization and washing reagent. Click-It reactions were carried out at room temperature for 30 min, after which the cells were washed with Click-iT^®^ saponin-based permeabilization and washing reagents. DNA was stained with Guava^®^ Cell Cycle Reagent (Cytek) for 30 min, and data were acquired using a Guava easyCyte flow cytometer.

### FUCCI cell line generation

Twenty-four hours prior to transduction, A549 or MDA-MB-436 cells were plated at 1 × 10^4^ cells/well in a 48-well plate. The Incucyte Cell Cycle Green/Red Lentivirus Reagent (Sartorius, Cat# 4779) was added at an MOI of 4.5 in media containing 8 µg/ml polybrene (Millipore Sigma, Cat# TR-1003). Beginning 48 h after viral transduction, the cells were subjected to puromycin selection at 0.6 µg/ml for 2‒4 weeks and then serially diluted in 96-well plates to select single-cell clones. Puromycin-resistant clones were selected based on the relatively equal intensities of GFP and RFP markers.

### Live cell imaging and analysis

For analysis of adherent live cells, cells were plated at 5 × 10^3^ cells/well in a 96-well plate and incubated for 18–24 h in a CO_2_ incubator at 37°C. Treatments are indicated in the figures. If indicated, Incucyte^®^ CytotoxRed Dye (Sartorius, Catalog #4632) was added at a final concentration of 0.25 µM at the time of drug treatment. The cells were transferred to the Incucyte imager after treatment and images were captured at 4× or 10× magnification at the indicated time intervals. Percent confluence was determined using Incucyte software applying a specific mask designed for each cell line. Uptake of the Cytotox Red dye was measured using the total integrated red fluorescence intensity.

### *In vivo* analysis

Female NOD-scid/IL2Rg-null (NSG) mice (*In Vivo* therapeutic core facility, IU Simon Comprehensive Cancer Center, Indianapolis, IN, USA) were housed in a pathogen-free facility at the IUSM LARC facility. Animal studies were approved by the Institutional Animal Care and Use Committee of the Indiana University School of Medicine. The hind flanks of 8–10-week-old NSG mice were implanted with MDA-MB-436 cells (~2.5 × 10^6^) in Matrigel. Tumor volume was monitored using electronic calipers [tumor volume = length × (perpendicular width)2 × 0.5]. Mice with tumors ranging from ~100 mm^3^ in size were randomized into individual treatment arms. RPAi NERx-329 was formulated in 5% NMP, 60% PEG300, and 30% Tween 80 and administered via intraperitoneal (IP) injection at 25 mg/kg once daily. Olaparib was dissolved in PBS and administered via oral gavage once daily at a dose of 5 mg/kg. Tumor volumes were monitored as indicated, and the results were presented as the average tumor volume ± standard error of the mean for each group. The final tumor weight was determined from the excised tumors at the end of the experimental period. Numbers (*n*) for each experiment are presented in the figure legends.

## Results

### RPA impacts at the replication fork

**Figure 1.**
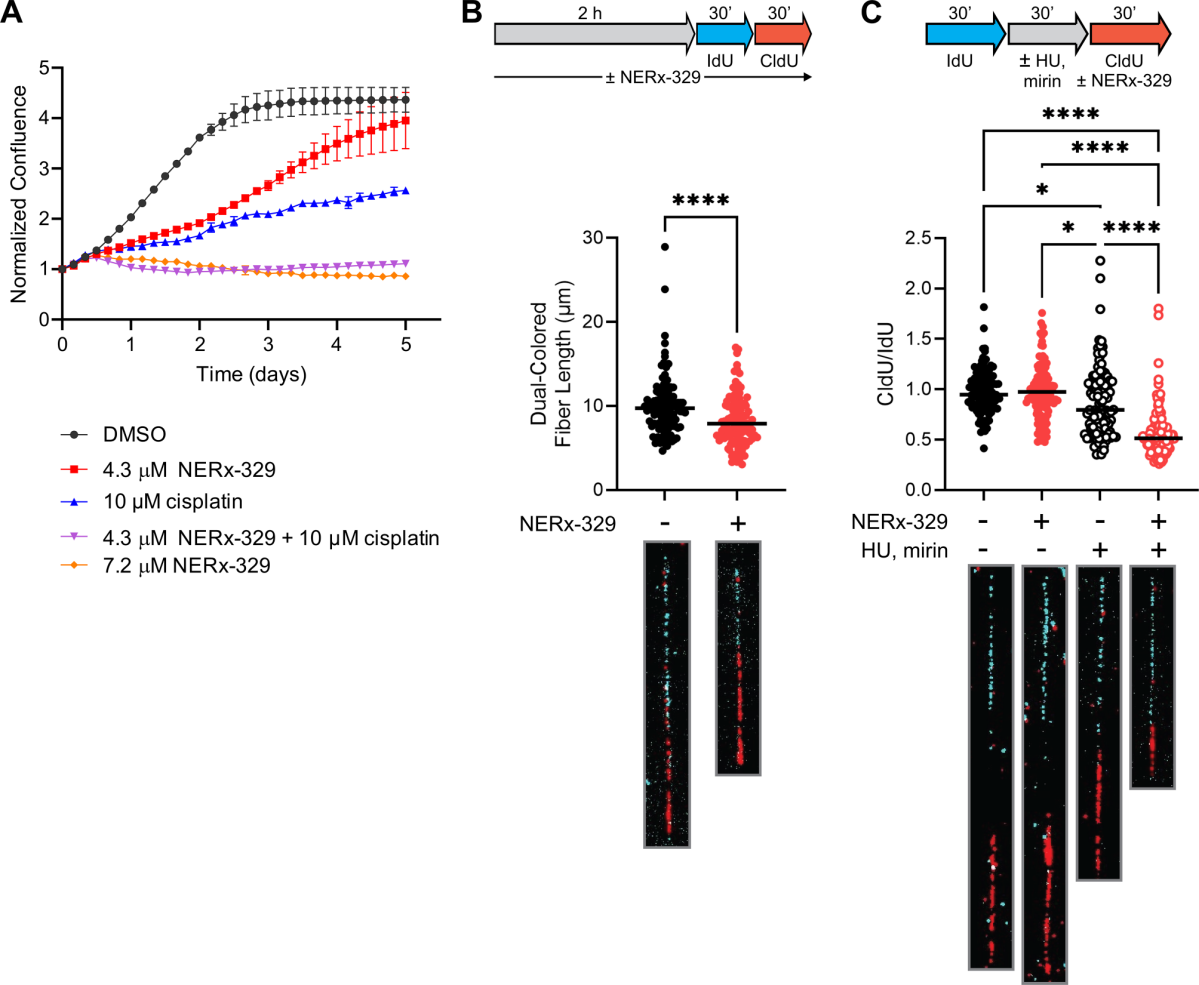
Chemical RPAi slows fork progression and inhibits replication restart in A549 NSCLC cells. (**A**) A549 cell proliferation was assessed via Incucyte live cell imaging as a function of NERx-329 and cisplatin treatment. The data are presented as the mean and SEM of triplicate determinations. (**B**) Replication fork progression in response to treatment with 30 µM NERx-329 was assessed by molecular combing using the scheme indicated. Data are presented as the median and individual values from at least 100 replication tracks from duplicate experiments. Representative tracks are indicated below the graph. Statistical analysis was performed via Student *t*-test ****P < .0001. (**C**) Replication fork restart in response to 4 mM HU, 50 µM mirin, and/or 30 µM NERx-329 was assessed by molecular combing using the scheme indicated. Data are presented as the median and individual values from at least 100 replication tracks from duplicate experiments. Representative tracks are indicated below the graph. Statistical analysis was performed via one way ANOVA (*P < .05, ****P < .0001).

We have previously demonstrated that chemical inhibition of RPA ssDNA binding activity enhances cisplatin anticancer activity *in vitro* and *in vivo* using first generation RPAi [8]. The increased biologic activity of more recent RPAi derivatives [7] has enabled a more thorough analysis of the cellular and molecular events impacted by RPAi treatment [9]. Live cell imaging reveals that treatment of A549 NSCLC cells with the RPAi NERx-329 results in a dose-dependent decrease in cellular proliferation (Fig. 1A) and that combination with cisplatin further reduces proliferation to the level observed with higher doses of RPAi. To determine the impact of RPAi treatment on events at the replication fork, we pursued single-molecule DNA fiber combing analyses. First, we treated A549 cells with RPAi for 2 h before sequential IdU and CldU labeling to assess replication fork progression. In an otherwise unperturbed S-phase, inhibition of RPA–ssDNA binding results in reduced fork length suggesting that RPA is necessary to maintain replication fork speed (Fig. 1B). RPAi treatment had no significant impact on other aspects of replication fork dynamics; the frequency of origin firing events and symmetry of bidirectional replication forks were unaffected (Supplementary Fig. S1A–C). HU-induced dNTP depletion results in fork stalling and was employed to allow the assessment of RPA function on replication fork restart. A549 cells were initially labeled with IdU before replication forks were briefly stalled with HU. RPAi treatment has previously been shown to result in the degradation of reversed forks upon prolonged HU [9], a process known to occur through the 3′-5′ exonuclease activity of MRE11. To avoid any confounding degradation effects on the measurement of fork restart, we therefore included the MRE11 inhibitor mirin during the fork stalling step to prevent replication fork degradation and shortening of IdU tracts in dual-colored DNA fibers. Replication was then allowed to resume in the presence or absence of RPAi and was labeled with CldU. RPAi treatment during the final 30-min DNA labeling had a negligible effect on fork progression while HU treatment resulted in fork stalling and restart after its removal as revealed by the decreased mean CldU/IdU ratio (Fig. 1C and Supplementary Fig. S1D–F). The presence of mirin had no significant effect on CldU/IdU ratios with or without NERx-329 treatment (Supplementary Fig. S1D and E). Interestingly, there are two distinct populations upon HU release; one population exhibited completely restored fork restart yielding a ratio of ~1 while the other population was impaired with a CldU/IdU ratio that was ~0.5. Blocking RPA binding during the restart period effectively slowed all restart events such that only a single population of replication events are evident, with a ratio below 0.5 (Fig. 1C) [20, 21]. Taken together, these results demonstrate that RPA ssDNA binding activity is important for replication fork progression and restart.

**Figure 2.**
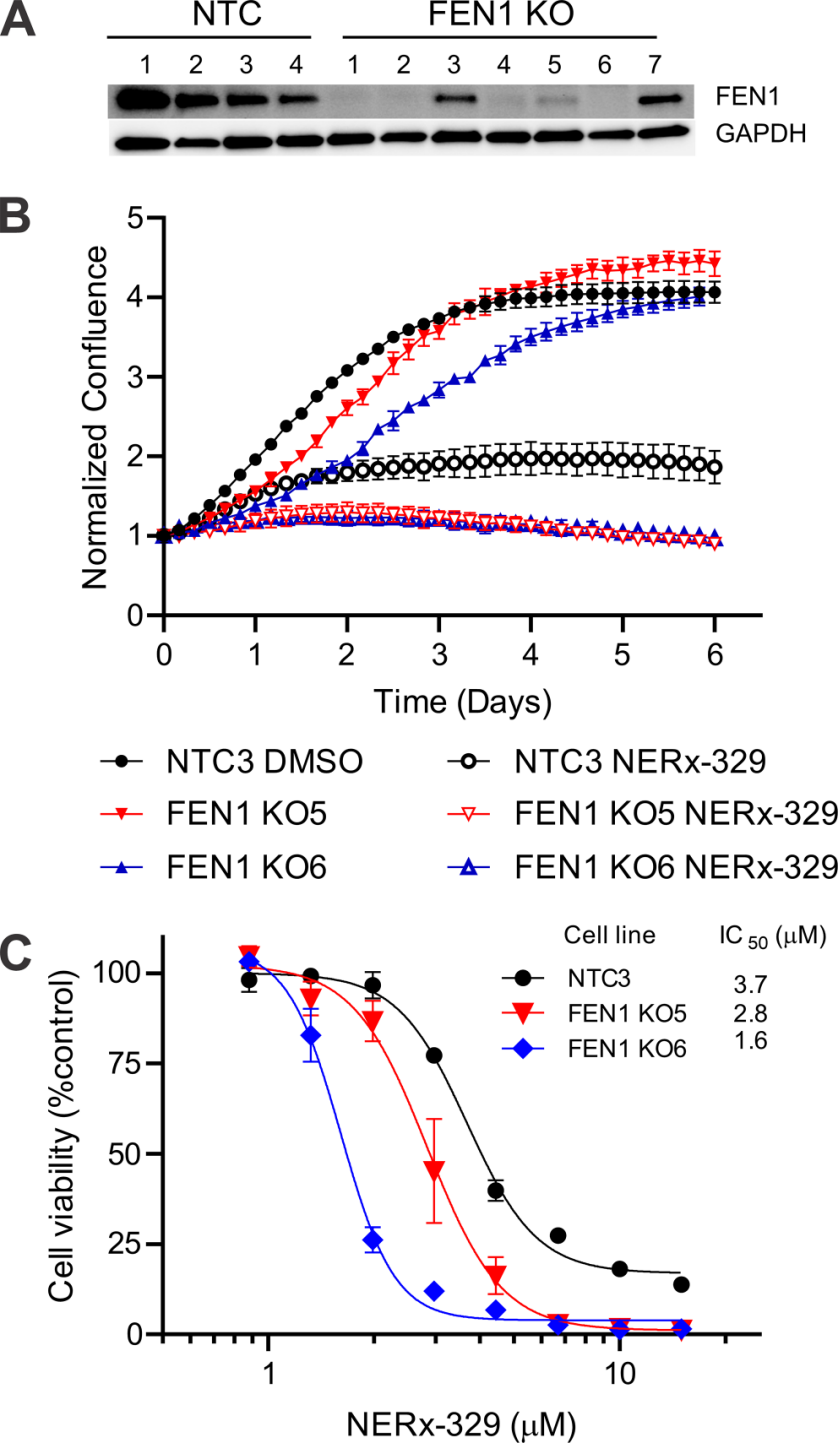
FEN1 loss sensitizes A549 NSCLC cells to RPAi. (**A**) Western blot confirming KO of FEN1 in A549 clonal cell lines. (**B**) Incucyte live cell imaging analysis of indicated A549 cells treated with 2.5 µM NERx-329. The data are presented as the mean and SEM of triplicate determinations. (**C**) Analysis of viability via CCK-8 metabolic assay in the A549 NTC, FEN1 heterozygous, and homozygous KO cells after treatment with RPAi NERx-329. The IC50 values were calculated by nonlinear regression analysis.

To gain insight into these molecular events driving RPAi-dependent toxicity, we conducted a CRISPR screen in A549 cells expressing endogenous Cas9 and introduced individual single guide RNAs to 229 DDR genes (Supplementary Table S1) with four guides per gene performed in triplicate. Twenty-four hours post transfection, cells were treated for 48 h with 2 µM NERx-329, a sub-toxic dose, to allow identification of potential synthetic lethal interactions. A reduction in proliferation of greater than 20% per guide was considered positive and the number of positive hits per gene determined. The top 17 genes (Supplementary Table S2) spanned various DNA replication, repair, and signaling pathways. The top gene identified was flap endonuclease 1 (FEN1), which encodes a protein involved in Okazaki fragment processing and its dysregulation results in increased ssDNA on the lagging strand [22, 23]. We used CRISPR/Cas9 gene editing to generate homozygous (FEN1 KO6) and heterozygous (FEN1 KO5) FEN1 knockouts in A549 NSCLC cells (Fig. 2A and Supplementary Fig. S2A). Live cell imaging revealed that the partial and complete loss of FEN1 resulted in a proportional decrease in growth rate compared to nontargeted control (NTC) cells (Fig. 2B). Treatment with RPAi revealed that the FEN1 KO cells were more sensitive to RPAi treatment, suggesting that the increase in ssDNA accompanied by FEN1 loss increases the reliance on RPA for protection of the ssDNA. Analysis of viability via CCK-8 metabolic activity assay also revealed that NERx-329 treatment resulted in a 2.3-fold change in IC_50_ in the NTC compared to the FEN1 homozygous KO cells (Fig. 2C). Consistent with these results, FEN1 loss resulted in a concomitant sensitization to the PARPi olaparib, which induces ssDNA gap formation (Supplementary Fig. S2B–D) [14]. These data confirm the CRISPR screen hit and also support the premise that increased ssDNA increases the reliance on RPA and renders cells more sensitive to RPAi. Further inspection of the hits identifies DSB repair in addition to S-phase replication as contributing to RPAi sensitivity. This is consistent with our previous demonstration that RPAi was moderately synergistic with PARP inhibition in DSB repair proficient NSCLC and more so in a noncancer RPE genetic model of BRCA1 deficiency [14].

### *In vivo* anticancer activity of the RPAi‒PARPi combination therapy

**Figure 3.**
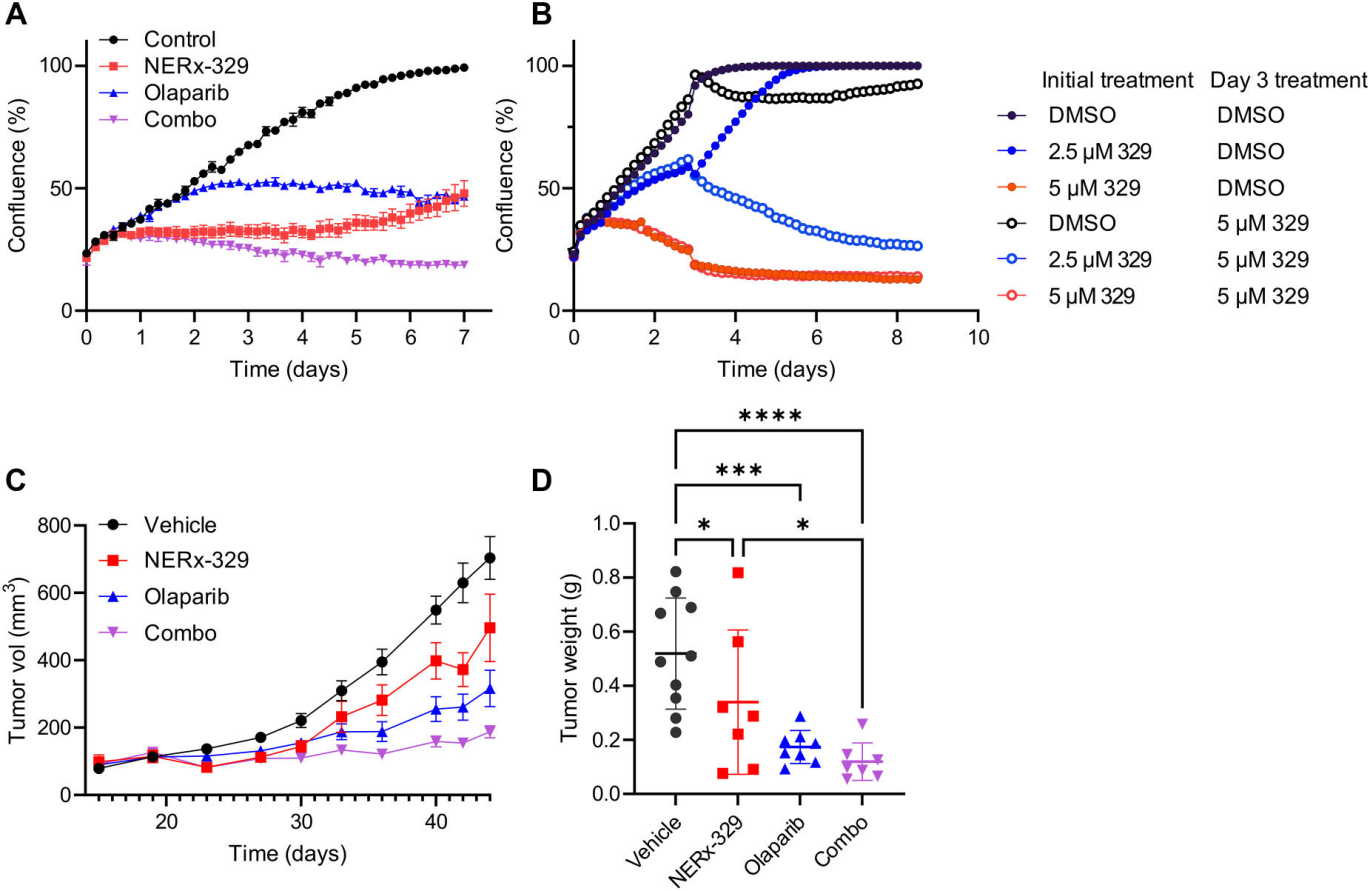
*In vivo* efficacy of RPAi combination treatment of BRCA1-deficient TNBC. (**A**) Live cell imaging analysis of RPAi combination treatment in MDA-MB-436 cells treated with 1 µM olaparib and/or 2 µM NERx-329. The data are presented as the mean and SEM of triplicate determinations. (**B**) Repeat dosing of RPAi and analysis via live cell imaging of MDA-MB-436 cells treated as indicated. The data are presented as the mean and SEM of triplicate determinations. (**C**) *In vivo* efficacy of RPAi combination therapy. MDA-MB-436 cells were implanted, and the tumor volume was measured. The indicated agents were administered via IP injection daily. (**D**) Terminal tumor weights from the experiment described in panel (C). The mean ± SEM is indicated, and the data were analysed using one-way ANOVA with Fisher’s LSD test; *P < .05, **P < .01, ***P < .001, ****P < .0001.

To better understand the impact of RPA protection of ssDNA, we employed the MDA-MB-436 BRCA1-deficient triple-negative breast cancer (TNBC) model. As both BRCA1 and PARP are potent suppressors of ssDNA gaps, we anticipated that in a bona fide cancer model, an increased reliance on RPA and hence sensitivity to RPAi treatment. We have previously demonstrated RPAi synergize with PARPi in BRCA1-deficient noncancerous models using colony formation assays [14]. To further determine whether combination PARPi-RPAi treatment could be an effective therapeutic strategy we employed live-cell imaging to assess cell growth. Olaparib did not affect proliferation until 48 h after treatment, after which proliferation was reduced (Fig. 3A), which is consistent with ssDNA gaps induced during the initial S phase persisting through the cell cycle and forming DSBs in the second S phase [17]. RPAi-mediated inhibition of proliferation was evident within 24 h, and cell confluence remained stable for ~4 days, after which an increase was observed (Fig. 3A). The efficacy of the single agent treatments was greatly enhanced in the combination, which resulted in essentially no growth observed through the 7-day assay and an actual reduction in confluence, indicative of cell death and not simply a reduction in proliferation (Fig. 3A). This implies that the PARPi-induced S-phase ssDNA gaps that can persist through the next cell cycle are instead degraded in the initial S phase when RPA protection is concurrently inhibited, resulting in replication catastrophe and cell death. The recovery of growth after ~4 days of treatment with the single agent RPAi treatment suggests either the emergence of resistance or a drug metabolism effect. To distinguish between these possibilities, we performed a similar experiment but added a second drug administration on day 3 (Fig. 3B). The decrease in confluence after the second RPAi dose demonstrates that the cells remain sensitive to RPAi treatment despite the initial resumption of cell growth, and are thus not acquiring RPAi resistance. These results and our previous *in vitro* analyses indicate that the combination of NERx-329 and olaparib could be an effective *in vivo* therapeutic strategy. To determine whether we could safely administer PARPi–RPAi treatment, we used MDA-MB-436 models *in vivo* that were subcutaneously implanted into NSG mice. When the tumors reached ~100 mm^3^, they were randomized to one of the four arms for single-agent or combination treatment. Olaparib and NERx-329 were both administered daily by oral gavage and IP, respectively. We did not observe any increased toxicity in the combination-treated mice compared to that in the NERx-329-treated mice, as assessed by body weight measurements (Supplementary Fig. S3). The data presented in Fig. 3C for assessing tumor volume over time show the efficacy of the combination, which abrogates tumor growth and thereby recapitulates the live-cell imaging data. The final tumor weight was determined, and the efficacy of the combination treatment was confirmed (Fig. 3D). These data demonstrate the utility of the combination of PARPi–RPAi therapy in BRCA1-deficient cancers by exploiting RPA protection capacity.

### RPA protects PARPi-induced ssDNA gaps in BRCA1-deficient TNBC cells

**Figure 4.**
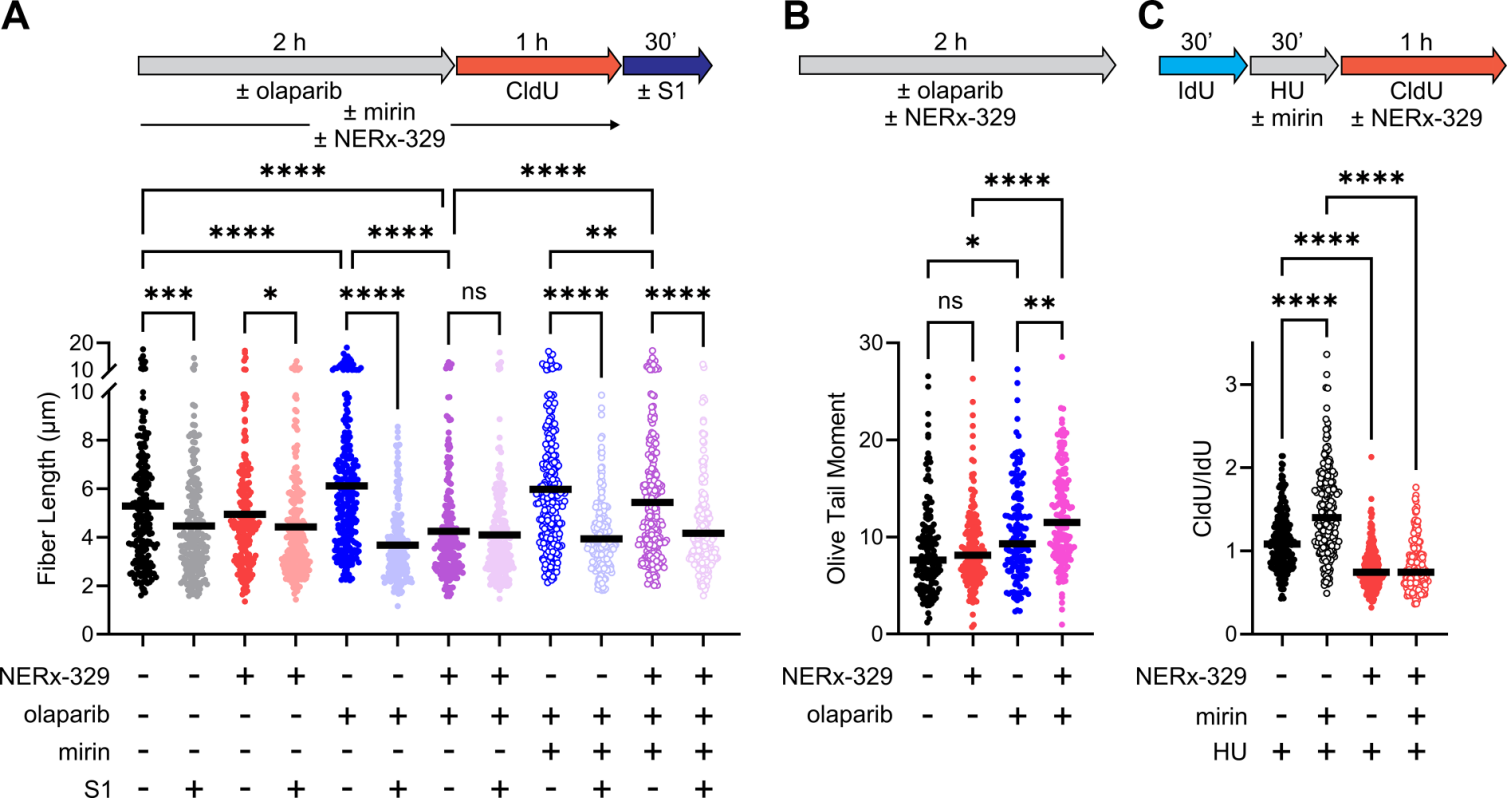
The effect of RPAi on PARPi-induced replication fork dynamics. (**A**) Assessment of ssDNA gap formation was performed by molecular combing as depicted in the scheme. MDA-MB-436 cells were treated as indicated with 10 µM olaparib, 30 µM NERx-329, and/or 50 µM mirin. (**B**) Olive tail moment from neutral comet assays of MDA-MB-436 cells treated with 10 µM olaparib and/or 30 µM NERx-329. (**C**) Replication fork restart in BRCA1-deficient MDA-MB-436 cells treated with 4 mM HU, 50 µM mirin, and/or 30 µM NERx-329. Data in all panels are presented as the median and individual values. Panels (A) and (C) depict at least 200 replication tracks from duplicate experiments. Panel (B) depicts at least 150 comets scored from triplicate experiments Statistical analysis was performed via one way ANOVA (*P < .05, **P < .01, ***P < .001 ****P < .0001).

To assess the underlying mechanism of this RPAi–PARPi therapeutic combination, we employed the BRCA1-deficient MDA-MB-436 TNBC model and DNA fiber combing combined with S1 nuclease treatment to determine its impact on replication fork dynamics and ssDNA gap formation [22, 24]. Following 2 h of individual or concurrent combination treatment, replication fork progression was monitored by the incorporation of CldU into DNA, which was then combed onto coverslips and detected using a fluorescently labeled antibody (Fig. 4A). Within each replicate, the collected cells were split into two fractions and processed identically to treat the released DNA with and without S1 nuclease immediately before being combed onto coverslips. S1 nuclease nicks ssDNA; thus, DNA fiber length shortening after S1 treatment is indicative of the presence of ssDNA gaps [25]. Consistent with BRCA1-deficient gap formation [13], control cultures treated with vehicle showed ssDNA gaps, as evidenced by a small decrease in DNA fiber length upon DNA fiber processing with S1 nuclease. Treatment with RPAi did not significantly affect DNA fiber length contrary to the results obtained in the BRCA wild type A549 cells, while ssDNA gaps were still evident in RPAi treated cells. As BRCA1-deficiency results in the loss of fork restraint in response to other DDR-targeted therapies (i.e. PARPi) and could potentially explain this difference [14], we employed the BRCA1-deficient UWB1.289 cell line and its BRCA1-complemented counterpart. Like A549 cells, UWB1.289 cells exhibited shorter DNA fibers and thus slowed replication in response to NERx-329 treatment, however DNA fiber lengths from BRCA1-complemented UWB1.289 cells were insensitive to NERx-329 treatment (Supplementary Fig. S4A), suggesting that the differential impact of NERx-329 on replication fork progression between A549 and MDA-MB-436 cells is related to other genetic factors outside of BRCA1-deficiency.

As expected, cells treated with olaparib exhibited accelerated replication and significantly increased DNA fiber lengths with extensive ssDNA gap formation [14, 26] (Fig. 4A). Strikingly, the combination of NERx-329 and olaparib reversed the apparent acceleration of replication and resulted in significantly shortened DNA fibers that were insensitive to S1 nuclease (Fig. 4A). These data suggest that either PARPi-induced fork acceleration is dependent on RPA function or that loss of RPA altered fork stability, similar to the defects in DNA replication observed in BRCA1 wild-type NSCLC A549 cells, though at a much greater magnitude (Fig. 1), or that the lack of ssDNA gaps could be a result of gap degradation due to the loss of RPA protection upon complete chemical RPA exhaustion. In this second scenario, the ssDNA gaps are degraded in the cell and converted to DSBs before any DNA fiber assay processing, and thus are insensitive to S1 nuclease.

To address this hypothesis, we included the MRE11 inhibitor mirin in treatments with olaparib alone and in combination with NERx-329. MRE11 is a critical nuclease responsible for expanding and degrading replication-dependent ssDNA gaps [27]. In addition, Seppa *et al*. recently observed that *in vitro*, MRE11 dependent gap cleavage does not occur unless there is loss of ssDNA protection by RPA, which we hypothesized would occur upon RPAi treatment [19]. Our results show that similar to treatment with olaparib alone, treatment with olaparib and mirin resulted in the formation of longer fibers that still contained ssDNA gaps. Importantly, the addition of mirin to MDA-MB-436 cells treated with olaparib and NERx-329 resulted in longer DNA fibers than the combination without mirin, and still resulted in ssDNA gaps, although the DNA fiber lengths were not completely rescued by co-treatment with mirin (Fig. 4A). These data support the model for the loss of ssDNA gap protection upon RPAi treatment, resulting in nucleolytic degradation into DSBs. Consistently, RPAi–PARPi combination treatment of MDA-MB-436 cells resulted in an increased Olive tail moment in neutral comet assays relative to either single agent treatment, indicative of DSB formation (Fig. 4B). Collectively, these data demonstrate that RPA is essential for the protection of PARPi-induced ssDNA gaps from MRE11-dependent degradation in BRCA1-deficient cells. Moreover, these data illustrate the RPAi therapeutic window as neither RPAi nor PARPi single-agent treatment completely exhausts RPA, as evidenced by the presence of ssDNA gaps after chemically lowering the quantity of active RPA or inducing ssDNA, respectively (Fig. 4A). However, the combination data are consistent with exhausted RPA such that ssDNA gaps are degraded and thus no longer detectable.

To assess the impact of RPA exhaustion on replication fork restart in BRCA1-deficient cells, MDA-MB-436 cells were first grown with IdU for 30 min, then replication was stalled with the addition of HU for 30 min, and replication was resumed in the presence of CldU with and without NERx-329 for 1 h (Fig. 4C). The additional 30-min incubation allows restart to be monitored efficiently as BRCA1 loss negatively impacts fork protection and restart [28, 29]. As both gap and fork degradation are dependent upon MRE11 nuclease activity, HU stalling was conducted in the presence and absence of mirin to ensure accurate measurement of replication fork restart as opposed to fork protection (Fig. 4C). The results demonstrate that mirin enhances fork restart and increased the CldU/IdU ratio (Fig. 4C). Interestingly, similar to A549 cells, two distinct populations are observed, mirin insensitive that displays a ratio of ~1 and the mirin sensitive where replication restart has occurred and the ratio is ~1.7, closer to the expected value of 2.0 if 100% restart was observed. Independent of mirin treatment, RPAi yielded fibers with a decreased CldU/IdU ratio, indicative of impaired replication fork restart (Fig. 4C and Supplementary Fig. S4B). The finding that mirin partially reverses RPA-dependent gap protection (Fig. 4A), yet does not rescue RPAi dependent inhibition of fork restart, suggests that RPA-dependent restart involves mechanisms other than gap protection. These effects could be a function of RPA’s role in supporting fork regression via SMARCAL1 and the coordinated regulation via post-translational modification ubiquitylation [20, 21].

Taken together, these data suggest that the amount of free RPA in BRCA1-deficient MDA-MB-436 cells was sufficient to protect the extensive ssDNA gaps generated by olaparib treatment from nuclease degradation. However, combination treatment with NERx-329 exhausts RPA and inhibits ssDNA protection such that the abundant ssDNA gaps are degraded by MRE11. Notably, cells treated with RPAi alone still possessed ssDNA gaps (Fig. 4A); therefore, the tested dosage and treatment duration did not completely exhaust RPA in the absence of additional RS-inducing agents such as olaparib. In contrast, olaparib treatment combined with NERx-329 yielded DNA fibers resistant to S1 nuclease (Fig. 4A), indicating complete RPA exhaustion by the combination of chemical inhibition and unrestrained ssDNA gap formation and degradation, that ultimately results in DSB formation (Fig. 4B). These data support the existence of a RPAi therapeutic window for cancer treatment afforded by oncogene- and/or DDRi-induced RS. Moreover, they describe a mechanism for the enhanced killing effect observed in NERx-329 and olaparib combination-treated BRCA1-deficient cancer cells. We hypothesized that enhanced killing was a result of genomic instability through replication fork collapse and chromothripsis, as has been observed under other treatment combinations that generate excessive ssDNA and/or exhaust RPA pools [5, 30, 31].

**Figure 5.**
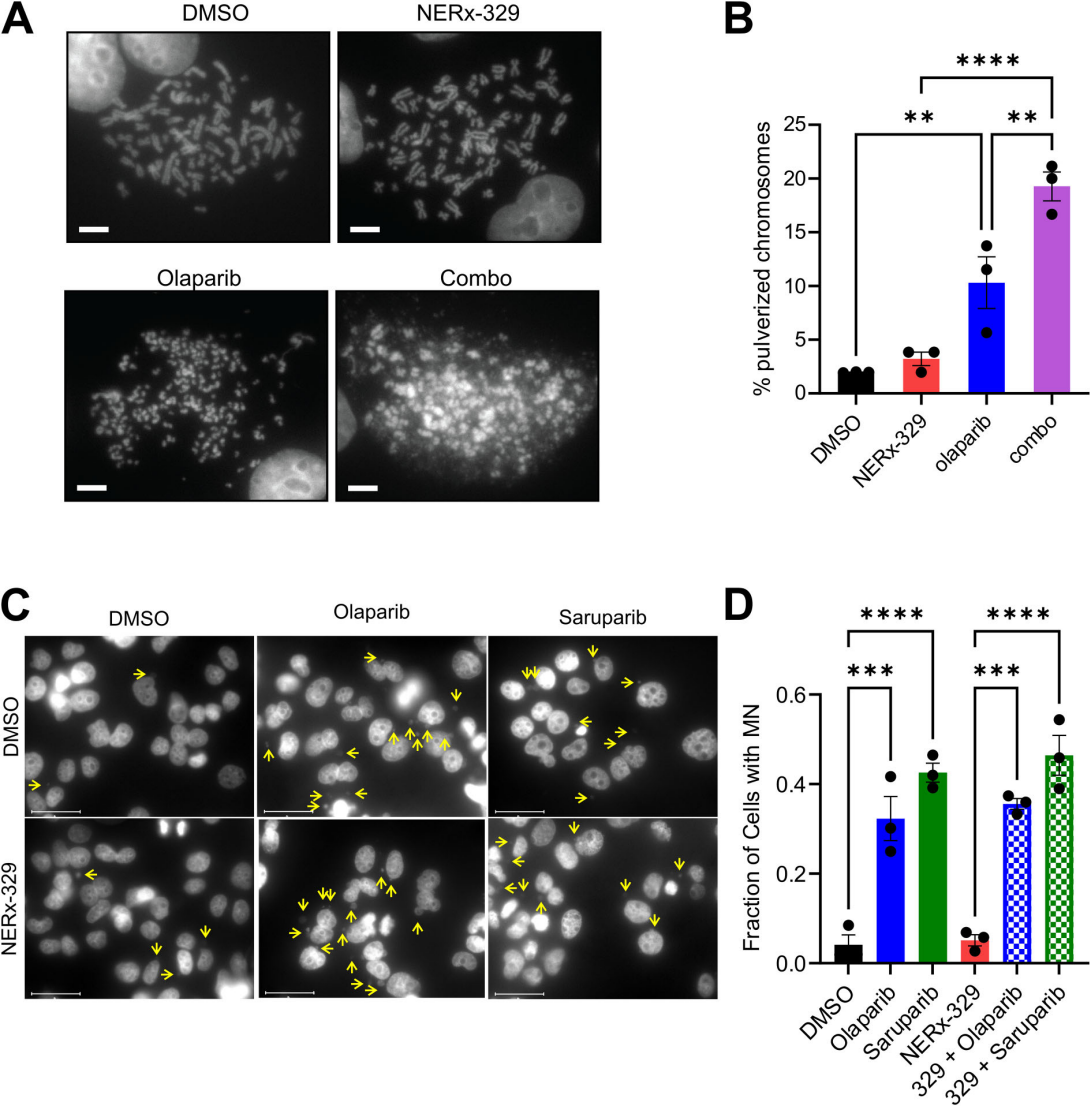
Effect of RPAi combination treatment on genomic instability. (**A**) Representative metaphase spreads from MDA-MB-436 cells treated with 2 µM olaparib and/or 5 µM NERx-329. (**B**) Quantification of pulverized chromosomes from the conditions depicted in panel B (*n* = 50 chromosomes/replicate; mean of triplicate experiments ± SEM). (**C**) Representative analysis of MN formation following treatment of MDA-MB-436 cells with 0.5 µM olaparib, 3 nM saruparib, and/or 1.5 µM NERx-329. (**D**) Quantification of MN formation in MDA-MB-436 cells from panel (C). The mean values are presented from triplicate independent experiments ± SEM quantifying 100 cells/replicate. All data were analyzed using one-way ANOVA with Fisher’s LSD test, *P < .05, **P < .01, ***P < .001, ****P < .0001.

To test this hypothesis, we performed metaphase spreads on single agent- and combination agent-treated MDA-MB-436 cells. Live-cell imaging experiments were performed to assess cell growth kinetics during single-agent and combination treatments to determine the experimental timing for the detection of chromosome pulverization before significant levels of cell death were observed (Supplementary Fig. S5). The results demonstrated that sequential treatment with olaparib for 2 days followed by NERx-329 for an additional day had a modest impact on growth on day 3 but completely inhibited growth by day 5. We employed this treatment scheme, and the cells were collected for the analysis of metaphase spreads on day 3 post-treatment (Fig. 5A). Single-agent RPAi treatment had no observable effect on the chromosome structure, whereas olaparib treatment increased chromosome pulverization. However, the combination strikingly induced chromosome pulverization (Fig. 5B). Taken together, NERx-329 chemically exhausts RPA such that olaparib-induced ssDNA gaps are degraded, and chromosomal integrity is compromised.

Considering the induction of chromosome pulverization, we assessed the generation of MN following RPAi treatment in combination with olaparib and the PARP1-specific PARPi saruparib. The quantity of MN in asynchronous cells was analyzed via DAPI staining, and the percentage of cells with at least 1 MN was quantified. The data revealed that PARPi treatment resulted in a significant increase in MN, whereas treatment with RPAi alone did not affect MN formation (Fig. 5C). The combination of RPAi–PARPi treatment did not significantly alter MN occurrence (Fig. 5D). Taken together, these results indicate that MN formation is not required for the enhanced cell killing effect of the combination treatment.

### PARPi specificity impacts RPAi efficacy

**Figure 6.**
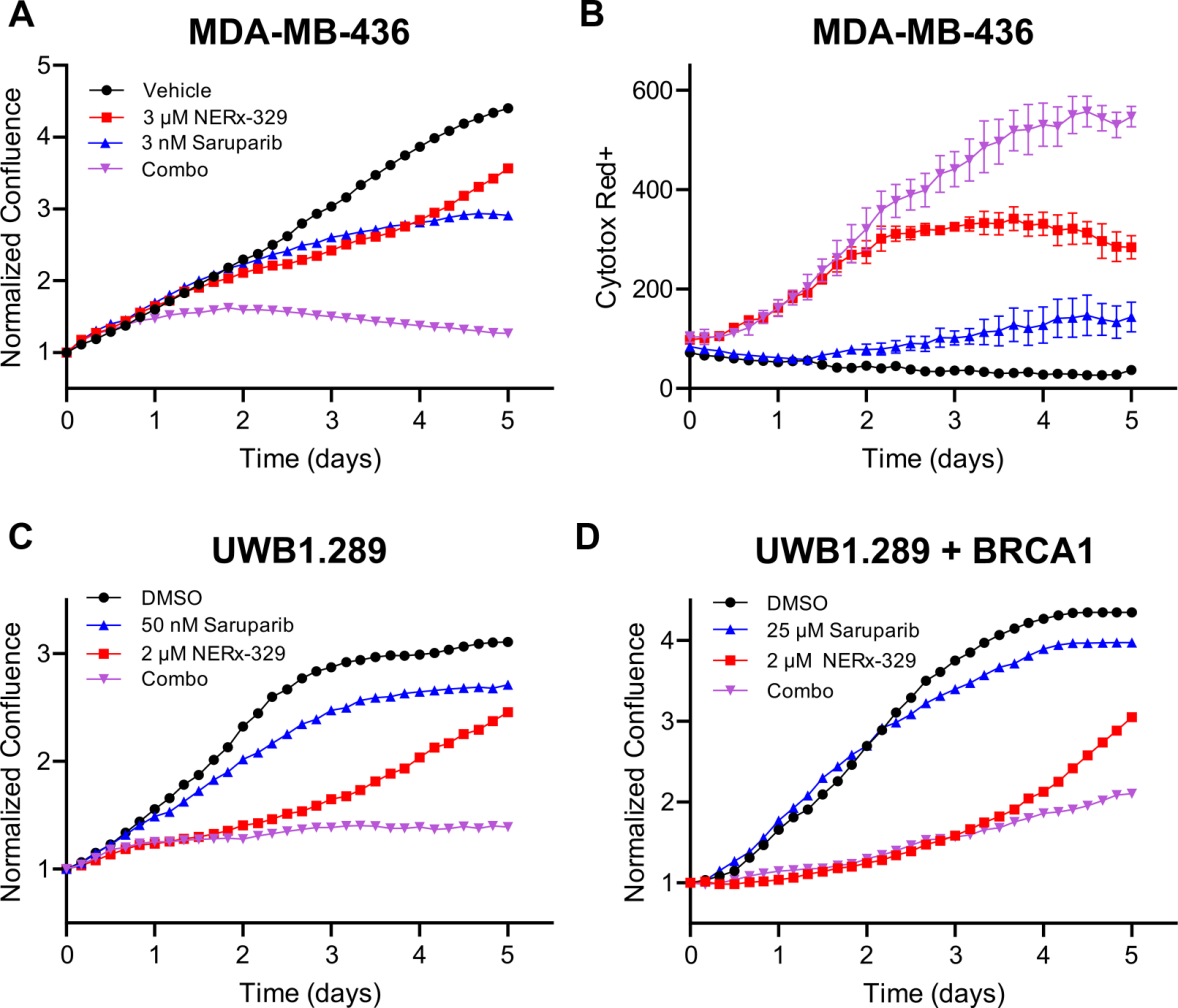
PARP1 specificity dictates PARPi - RPAi combination response. (**A**) Confluence of MDA-MB-436 cells treated with vehicle control, NERx-329, saruparib, or their combination was monitored by Incucyte live-cell imaging. (**B**) Cytotox Red dye fluorescence intensity from the experiment depicted in panel (A). (**C**) Confluence of UWB1.289 cells treated with the vehicle control, NERx-329, saruparib, or a combination, as monitored by Incucyte live-cell imaging. (**D**) Confluence of BRCA1-complemented UWB1.289 cells treated with vehicle control, NERx-329, saruparib, or a combination, as monitored by Incucyte live-cell imaging.

As our mechanistic studies of ssDNA gap protection translated into a positive *in vivo* therapeutic combination with olaparib, we sought to determine how PARPi specificity affected the combination treatment, as olaparib is an inhibitor of both PARP1 and PARP2. To assess specificity, we employed the more potent and selective PARP1-specific inhibitor, saruparib (AZD5305) [32]. Considering the accurate recapitulation of *in vivo* antitumor activity with 5-day live-cell imaging analyses, we assessed saruparib in combination with NERx-329 in the BRCA1-deficient MDA-MB-436 TNBC cell line using this methodology. Similar to the results obtained with olaparib, the combination of RPAi with the PARP1-specific inhibitor saruparib resulted in virtually no growth and induced cell death, as indicated by the uptake of Cytotox Red dye (Fig. 6A and B), potentially due to the activation of caspase 3/7 as has been shown for single-agent NERx-329 treatment [9]. In addition, only minor growth defects were observed when treating BRCA1-deficient cells with NERx-329 in combination with the PARP2-specific inhibitor UPF1069, and synergy was specifically driven by saruparib in the triple combination (Supplementary Fig. S6A). These data demonstrate that the synergistic effects of chemical RPAi result from PARP1 inhibition. To ensure that this was not a cell-model-specific event, and to determine the impact of BRCA1 status, we assessed the PARPi-RPAi interaction in the isogenic UWB1.289 ovarian cancer model. In BRCA1-deficient UWB1.289 cells, the maximal activity of saruparib was observed at 50 nM (Fig. 6C); a 10-fold increase to 500 nM had no additional effect on cell proliferation over the 7-day experiment (Supplementary Fig. S6B). NERx-329 significantly inhibited cell growth over the first three days of treatment (Fig. 6C), after which proliferation resumed to a doubling time similar to that of untreated cells, as previously observed (Figs 1A and 3A). In the combination treatment, the cells remained nonproliferative throughout the 7-day experiment despite a single treatment at day 0 (Fig. 6C). These data support a model in which sufficient PARPi-dependent ssDNA is generated, such that RPAi combination treatment induces chemical exhaustion of RPA and is responsible for the observed cell death. The BRCA1-complemented cell line is a model of PARPi resistance and, as expected, is far less sensitive to PARPi saruparib; treatment at a dose of 25 µM had only a modest effect on cellular proliferation (Fig. 6D). Importantly, these PARPi-resistant cells were still sensitive to RPAi single-agent treatment, and the combination clearly displayed greater anticancer efficacy than either individual treatment (Fig. 6D). These data are consistent with PARPi induction of ssDNA gaps, independent of BRCA1 status, with BRCA1-proficient cells requiring higher doses or longer PARPi exposure than BRCA1-deficient cells [14, 26]. Moreover, these data support the hypothesis that the combined efficacy of PARPi and RPAi is derived from processes that generate ssDNA and are not confined to a BRCA1-deficient background, consistent with the sensitization observed in FEN1-deficient cells (Fig. 2).

### Impact of combination RPAi–PARPi treatment on cell cycle progression

**Figure 7.**
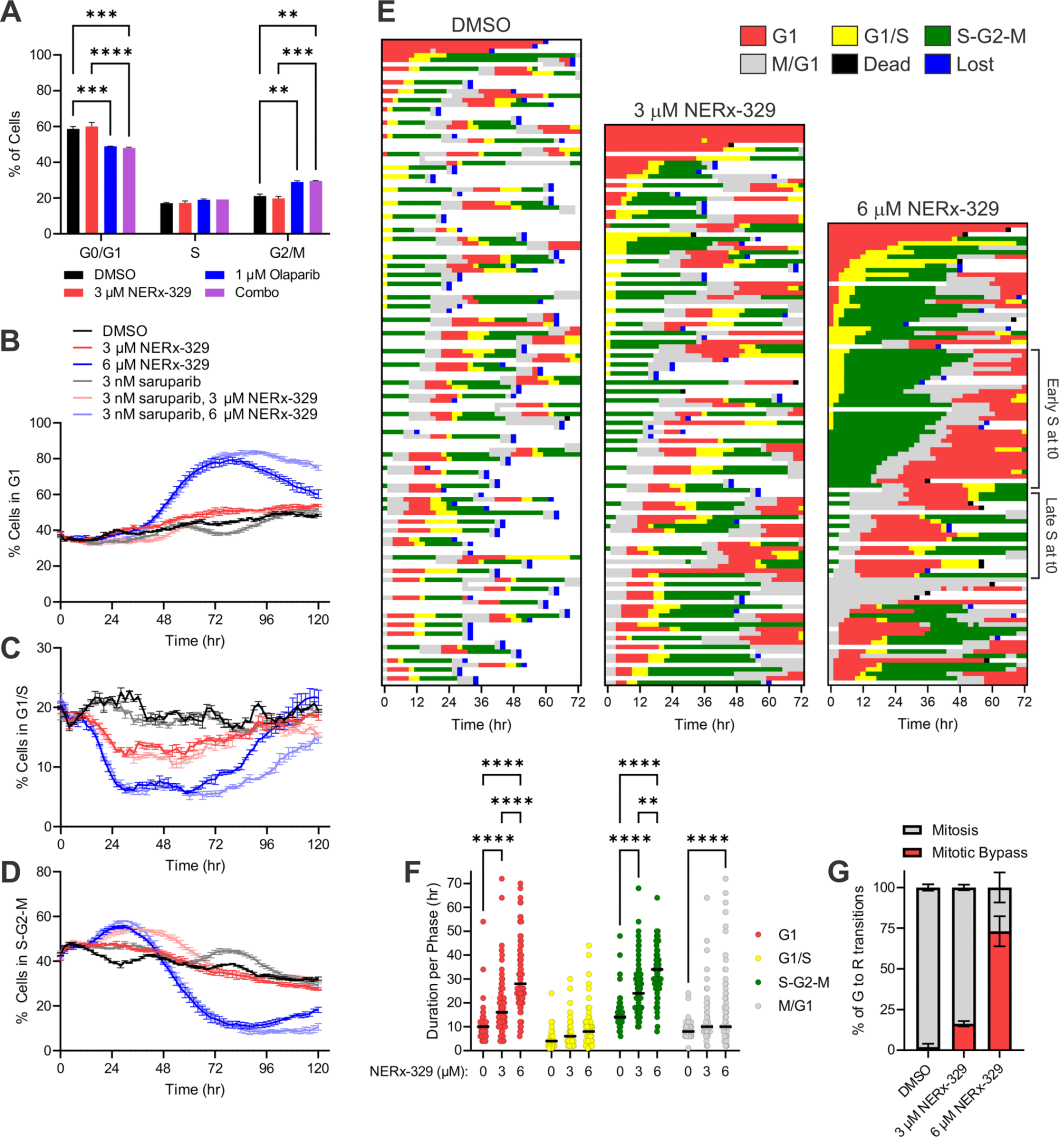
Effects of NERx-329 and PARPi combination treatment on the cell cycle. (**A**) Quantification of the cell cycle distribution was quantified in MDA-MB-436 cells treated with the vehicle control, NERx-329, olaparib, or their combination, as measured by flow cytometry (mean ± SEM). Quantification of MDA-MB-436 FUCCI cells in (**B**) G1, (**C**) G1/S, or (**D**) S-G2-M following treatment with vehicle control, NERx-329, saruparib, or their combination (mean ± SEM). (**E**) Individual MDA-MB-436 FUCCI cell trajectories following treatment with the vehicle control or increasing concentrations of NERx-329 (*n* = 54, 68, and 85 cells at the initial time points, respectively, from duplicate experiments). Single cells were followed until they were lost/untrackable owing to cell overlap (blue). (**F**) Quantification of MDA-MB-436 FUCCI cell cycle duration following treatment with vehicle control or increasing concentrations of NERx-329 (*n* = 66–112 from duplicate experiments). The median measurement is indicated by the solid black bar. (**G**) Quantification of MDA-MB-436 FUCCI cells that transitioned from green (G) to red (R) and successfully divided (mitosis) or engaged in the next cell cycle without dividing (mitotic bypass) following treatment with the vehicle control or increasing concentrations of NERx-329 (*n* = 34–50 transitions from duplicate experiments). The data were analysed using one-way ANOVA with Fisher’s LSD test; *P < .05, **P < .01, ***P < .001, ****P < .0001.

To better understand the downstream cellular effects of the loss of ssDNA gap protection that underlies the efficacy of the RPAi–PARPi combination *in vitro* and *in vivo*, we investigated its impact on cell cycle progression. Previous analyses of the predecessor RPAi demonstrated minimal effects on the cell cycle in BRCA wild-type cancers [33], and the results obtained in BRCA1-deficient MDA-MB-436 cells were very similar, with minimal impact observed as assessed by flow cytometry after 48 h (Fig. 7A). PARPi treatment in BRCA1-proficient cell lines results in the accumulation of cells in G2/M, with a reduction in the G1 population [26], whereas BRCA1-deficient ovarian cancer cells do not show this effect owing to a failure in checkpoint activation [17]. Similarly, we found that olaparib single-agent activity slightly increased the number of cells in G2/M phase and decreased the number of cells in G1 phase (Fig. 7A). All the observed changes in the cell cycle distribution at this 48 h timepoint were driven by PARPi, as combination treatment with RPAi and PARPi did not further affect the cell cycle distribution (Fig. 7A and B). To more accurately assess the effects on individual cells, we employed the fluorescent ubiquitin cell cycle imaging (FUCCI) system to monitor cell cycle progression in live MDA-MB-436 cells (Supplemental Movies S1 and S2, DMSO- and RPAi-treated cells, respectively). FUCCI cells exhibit red fluorescence during G1 (Cdt1-mKate2) and green fluorescence during S-G2-M (geminin-mTagGFP2) [34, 35]. The cells are fluorescent yellow at G1/S and colorless during division and initiation into the next cell cycle (M/G1). The growth of MDA-MB-436 FUCCI cells in response to NERx-329, saruparib, or combination treatment was similar to that of the parental MDA-MB-436 cell line (Supplementary Fig. S7A). We found that RPAi treatment had a biphasic effect on the cell cycle distribution of MDA-MB-436 FUCCI cells (Fig. 7B–D). The initial phase exhibited a modest increase in the S-G2-M population at the expense of the G1/S population over 48 h. The second phase exhibited a large accumulation of the G1 population (Fig. 7B), concomitant with a large reduction in the S-G2-M population (Fig. 7D). Both single-agent treatment and combination treatment with saruparib had little to no effect, and disruption of the cell cycle was driven by NERx-329 treatment in a dose-dependent manner (Fig. 7B–D and Supplementary Fig. S7B–E). Similar G1 accumulation was observed in NSCLC A549 FUCCI cells (Supplementary Fig. S7F–H), indicating that the effects of the cell cycle were independent of BRCA1 status. To further evaluate these RPAi-dependent effects, we tracked individual vehicle control- or NERx-329-treated MDA-MB-436 FUCCI cells for 72 h or until they could no longer be tracked owing to cell overlap (Fig. 7E). We found that NERx-329 treatment induced G1 and S-G2-M delays in a dose-dependent manner with a minor effect on M/G1 progression (Fig. 7E–F). Consistent with the induction of replication catastrophe, the prolonged G1 effect was dependent on RPAi treatment occurring in the previous S phase; MDA-MB-436 FUCCI cells in the M/G1 phase at the initiation of RPAi treatment did not exhibit delayed G1 durations compared with those starting in G1, G1/S, or S-G2-M, all of which would be required to go through the S phase before presenting a measurable G1 phase (Supplementary Fig. S7I). We also observed that a large proportion of MDA-MB-436 FUCCI cells treated with NERx-329 exhibited mitotic bypass by transitioning from green to colorless to red throughout the next cell cycle without successful division (Fig. 7G and Supplemental Movie S3). We did not observe evidence of abortive mitosis or binuclear formation in the G1 population of these cells, but rather an increased area of nuclear Cdt1-mKate2 expression after 72 h of treatment, likely representative of 2N DNA content (Supplementary Fig. S7J–M). Analysis of these results within the context of single-cell trajectories revealed that cells that were in the later stages of S-G2-M at the start of RPAi treatment (those that underwent mitosis in <10 h) were able to successfully divide, whereas those in the earlier stages of S-G2-M at the start of RPAi treatment (those that underwent mitosis in >10 h) exhibited a significantly increased frequency of mitotic bypass (Fig. 7E). These results are consistent with other reports describing S phase- and DDR-dependent induction of mitotic bypass in response to oncogenic RS or other DNA-damaging agents [36, 37].

## Discussion

**Figure 8.**
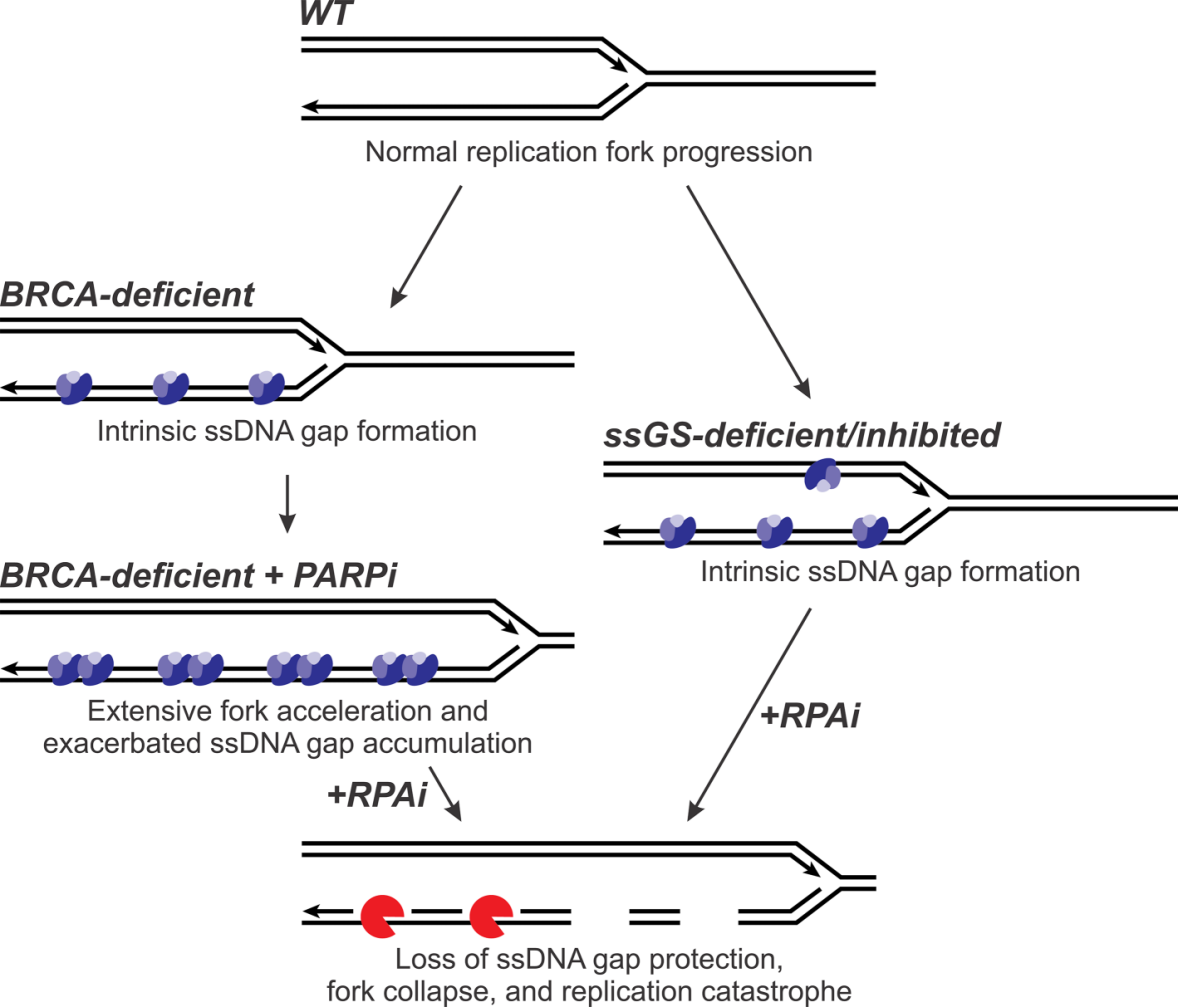
Model of chemical RPA exhaustion. RPAi–PARPi combination treatment efficacy is derived from the loss of ssDNA gap protection, of which gap formation is intrinsic to BRCA-deficient cells and exacerbated by PARPi treatment (left). RPAi combination treatment is therefore proposed to be analogously effective in other ssDNA gap suppressor (ssGS)-deficient or -inhibited contexts (right). See Supplementary Table S3 for additional putative ssGS genes.

Nearly two decades ago, synthetic lethality was first demonstrated using PARPi in BRCA-deficient cancer. Cell death has been attributed to the formation of DSBs via replication through unrepaired single-strand nicks in the template DNA, which cannot be repaired by HR owing to the deficiency of BRCA proteins. The discovery of PARP trapping activity as a driver of cellular sensitivity added to the additional caveat that PARPi induces a type of DNA damage through direct trapping of PARP proteins on DNA [38–40]. While the importance of BRCA protein-dependent processes is certainly an aspect of PARPi lethality [41], recent investigations have uncoupled PARPi sensitivity from HR/DSB repair and instead pointed to ssDNA gap accumulation as the initial driver of the therapeutic response [13, 14, 42]. In this model, ssDNA gaps have been proposed as the key therapeutic lesion-driving sensitivity, whereas DSBs are in part downstream manifestations resulting from apoptosis [14, 17]. While the absolute size of ssDNA gaps formed in cancer cells is not known and likely differs depending on drug combination and genetic background, ssDNA gaps have been observed to be between 100 and 2000 nt when induced in eukaryotic systems [19, 23, 43]. Moreover, the combination of multiple confounding factors (in our case, BRCA1 deficiency and PARPi treatment) increased the frequency of ssDNA gap occurrence and size [43]. These compounding effects can induce sufficient ssDNA gaps to sequester and exhaust all RPA, such that unprotected ssDNA leads to replication fork collapse and chromothripsis [5]; alternatively, the same result can be achieved via RPAi combination treatment and chemical RPA exhaustion (Fig. 8).

In this study, we delineated the underlying *in vivo* mechanism of the RPAi–PARPi combination in BRCA1-deficient cancer primarily as the loss of ssDNA gap protection. RPA participates in numerous DNA metabolic processes that may potentially impact RPAi therapeutic efficacy, including ATR signaling, replication fork remodeling, NER, and HR. While RPAi function in these pathways may dictate efficacy in other therapeutic contexts, our results demonstrate that the protection of ssDNA, specifically replication-associated ssDNA gaps, primarily drives RPAi synergy with PARPi. These studies demonstrate that the efficacy of PARP-targeted therapy is enhanced by depleting RPA and a lower ssDNA protection threshold (Fig. 8). Therefore, both treatments individually target the protection of ssDNA; PARPi induces ssDNA, and RPAi depletes RPA protection capacity. When combined, the mechanism of the synergistic interaction involves contributions from both BRCA1-deficiency and PARPi treatment, which generates excess ssDNA through the formation of lagging strand ssDNA gaps [12], and RPAi treatment, which sequesters free RPA, leading to RPA exhaustion (Fig. 8). In this context, RPAi treatment exploits specific vulnerabilities for anticancer action and offers the potential to reduce PARPi dosage to limit PARPi-associated off-target toxicity or overcome PARPi resistance, which is almost universally observed in patients with BRCA1-mutant HGSOC [44]. Moreover, the increased sensitivity to RPAi via ssDNA gap induction in three distinct backgrounds, deficiency of BRCA1 or FEN1, and inhibition of PARP1, suggests that other genetic alterations that affect replication fork dynamics and induce ssDNA gaps may also be susceptible to RPAi (Supplementary Table S3). RPAi functions through the same mechanism as described in this study to sequester free RPA alongside the ssDNA generated by therapeutic/deficiency (Fig. 8).

Therefore, this may allow for lower and more tolerable drug doses by minimizing off-target toxicities when used in combination with RPAi. Additionally, the identification of genetic factors that drive ssDNA gap formation could lead to the identification of novel therapeutic targets for combination studies. Moreover, potential NERx-329 therapeutic combinations provide novel alternatives to systems of acquired resistance, for example, PARPi resistance, as RPAi combination efficacy is not specific to PARP function, but rather to ssDNA formation and protection in general (Supplementary Table S3). Indeed, PARPi-resistant cells exhibit vast ssDNA generation owing to the expansion of nicks/SSBs, which can likely be leveraged for RPAi therapeutic efficacy [18]. Similarly, many other processes that increase ssDNA gap formation where RPAi is also predicted to trigger sensitivity [13, 14, 45–53]. Therefore, RPA exhaustion-driven cancer cell death may be useful in many cancers that arise from genomic instability initiated by mutations or alterations in genes involved in maintaining genomic integrity.

## Supplementary Material

gkag396_Supplemental_Files

## Data Availability

All data generated or analyzed during this study are included in this published article (and its supplementary information files).
