# Supplementary material for: Replication protein A protects lagging strand gaps, restricting PARP inhibitor-induced synthetic lethality in BRCA1-deficient tumors
Source: Nucleic Acids Res. 2026 Apr 28;54(8):gkag396. doi: 10.1093/nar/gkag396 (PMC13122181; doi:10.1093/nar/gkag396)
Supplement: gkag396_Supplemental_Files [file gkag396_supplemental_files.zip › Revised SuppTableLegends.docx]

**Supplemental Table Legends for:**

**Replication Protein A Protects Lagging Strand Gaps, Restricting PARP Inhibitor-Induced Synthetic Lethality in BRCA1-Deficicient Tumors**

Pamela S. VanderVere-Carozza*, Matthew R Jordan*, Joy E. Garrett, Karen E. Pollok, Katherine S. Pawelczak and John J. Turchi

**Supplemental Table Legends**

**Supplemental Table 1.** List of 229 DDR genes used in the CRIPSR screen.

**Supplemental Table 2.** NERx-329 CRISPR screen hits and scores.

**Supplemental Table 3.** List of reported proteins that result in ssDNA gap formation when dysregulated/inhibited and drugs/DNA damaging agents that induce ssDNA gaps, including the method of ssDNA gap detection, PMID, and DOI.
