# Supplementary material for: Replication protein A protects lagging strand gaps, restricting PARP inhibitor-induced synthetic lethality in BRCA1-deficient tumors
Source: Nucleic Acids Res. 2026 Apr 28;54(8):gkag396. doi: 10.1093/nar/gkag396 (PMC13122181; doi:10.1093/nar/gkag396)
Supplement: gkag396_Supplemental_Files [file gkag396_supplemental_files.zip › Supplemental Information-NAR-03.pdf]

Supplemental Information for:

Replication Protein A Protects Lagging Strand Gaps, Restricting PARP Inhibitor-Induced Synthetic Lethality in BRCA1-Deficient Tumors

Pamela S. VanderVere-Carozza*, Matthew R Jordan*, Joy E. Garrett, Karen E. Pollok, Katherine S. Pawelczak and John J. Turchi

Supplemental Figures

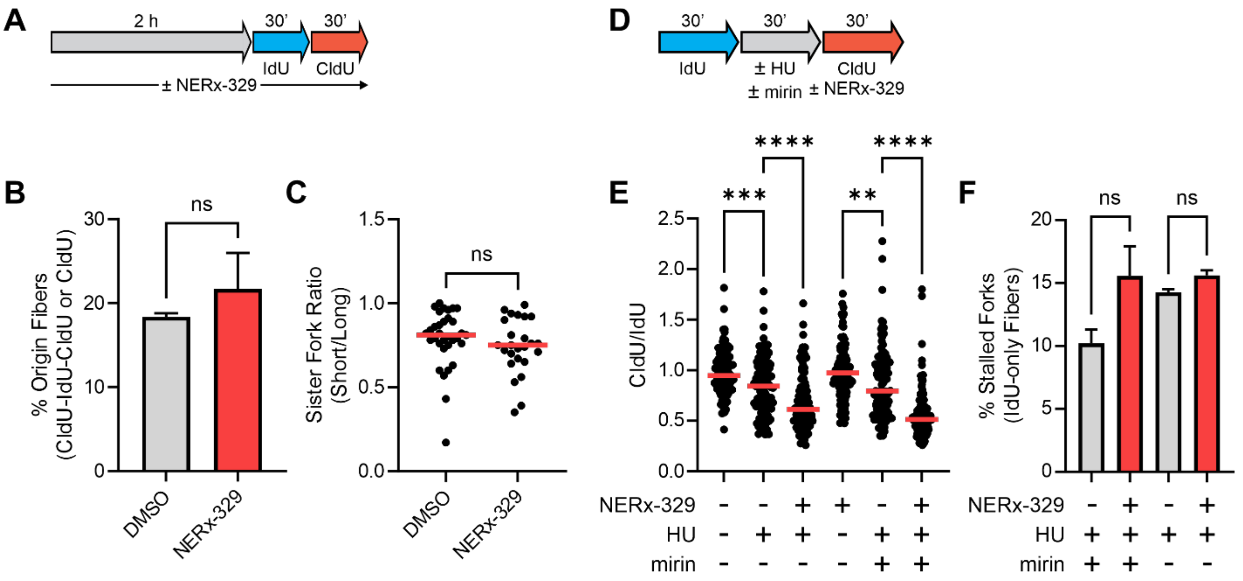

Figure S1. A) DNA fiber combing experiment labeling scheme for replication fork dynamics analysis upon treatment of A549 NSCLC cells with 30 µM NERx-329 for panels B-C. **B)** Quantification of origin firing events in response to NERx-329 treatment. **C)** Quantification of sister fork symmetry in response to NERx-329 treatment. **D)** DNA fiber combing experiment labeling scheme for replication fork restart analysis upon treatment of A549 NSCLC cells with 4 mM hydroxyurea (HU), 50 µM mirin, and/or 30 µM NERx-329 for panels E-F. **E)** Quantification of replication restart in response to HU, mirin, and/or NERx-329 treatment (Data partially reproduced from Figure 1C). **F)** Quantification of fork stalling in response to HU, mirin, and/or NERx-329 treatment.

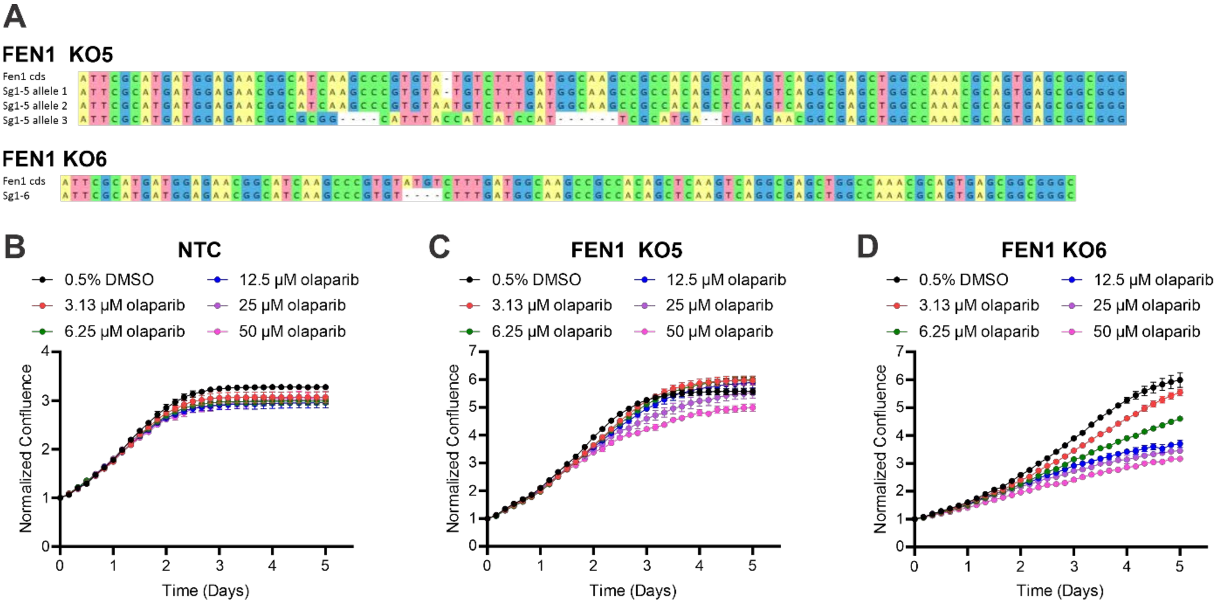

Figure S2. A) Sequencing results of the A549 heterozygous (FEN1 KO5) and homozygous (FEN1 KO6) CRIPSR knockout cell lines. Sensitivity of **B)** A549 NTC cells, **C)** A549 FEN1 KO5, and **D)** A549 FEN1 KO6 to increasing concentrations of olaparib.

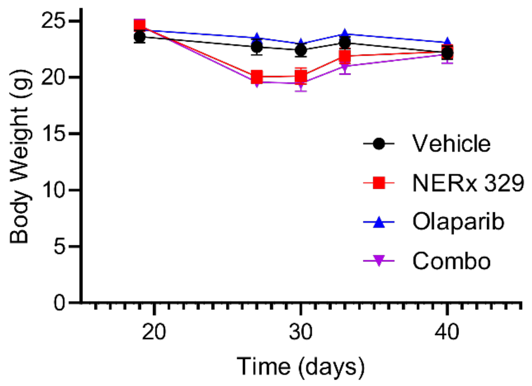

Figure S3. Toxicity assessment of RPAi/PARPi Combination therapy. Body weight was determined at the indicated time points in mice treated with the indicated agents as described in Figure 2.

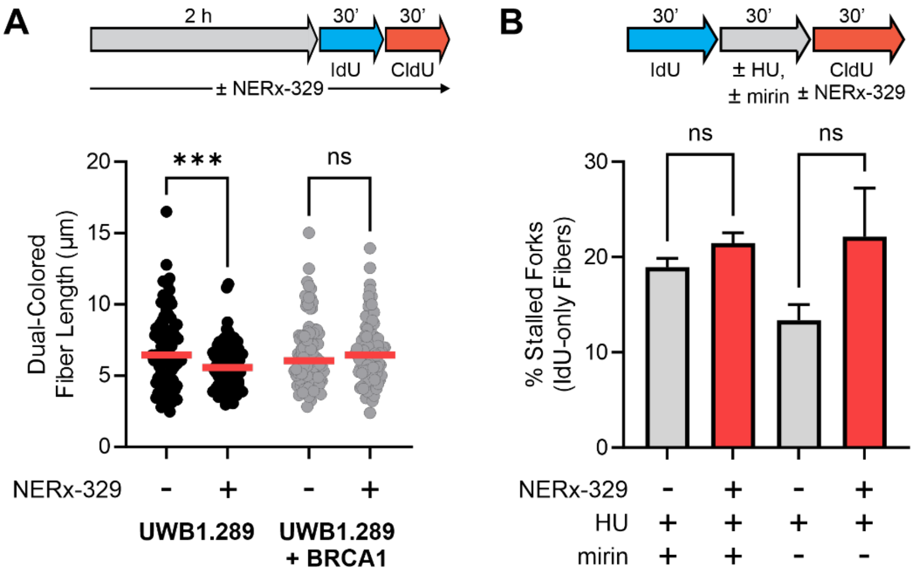

Figure S4. A) DNA fiber combing experiment labeling scheme for replication fork dynamics analysis upon treatment of UWB1.289 or UWB1.289 + BRCA1 cells with 30 µM NERx-329 and quantification of dual-colored fiber lengths. At least 100 DNA fibers were measured for each condition. **B)** DNA fiber combing experiment labeling scheme for replication fork restart analysis upon treatment of MDA-MB-436 TNBC cells with 4 mM hydroxyurea (HU), 50 µM mirin, and/or 30 µM NERx-329 for panels and quantification of fork stalling.

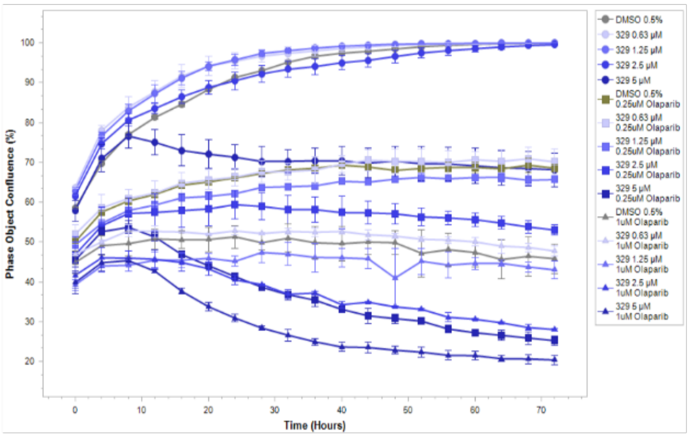

Figure S5. Confluence of MDA-MB-436 cells pretreated for 2 days with vehicle control or olaparib (0.25 or 1 μM), followed by the addition of vehicle control or NERx 329 (0.63, 1.25, 2.5, or 5 μM, Day 0 on the graph) as monitored by Incucyte live cell imaging. Olaparib pretreatment was conducted before beginning the live cell imaging.

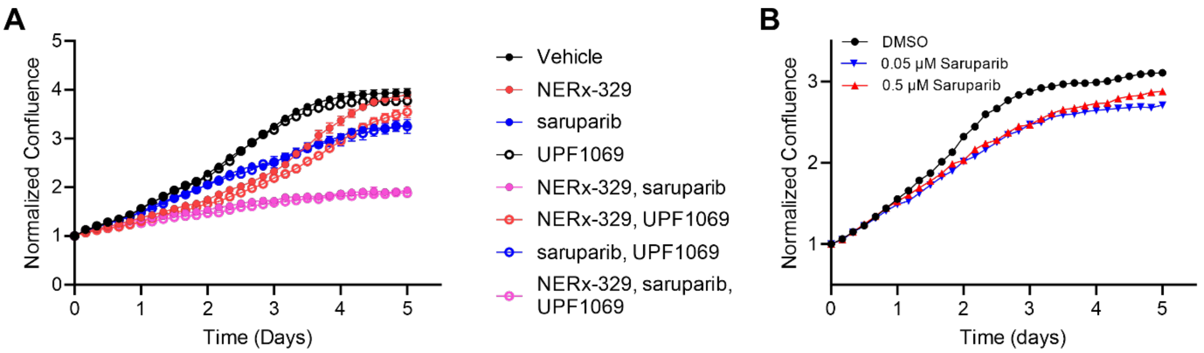

Figure S6. A) Live cell confluence of UWB1.289 cells treated with 1 μM NERx-329, 50 nM saruparib, and/or 10 μM UPF1069. **B)** Live cell confluence of UWB1.289 cells treated with increasing concentrations of saruparib. Confluence was monitored by Incucyte live cell imaging and normalized to the Day - 0 control images.

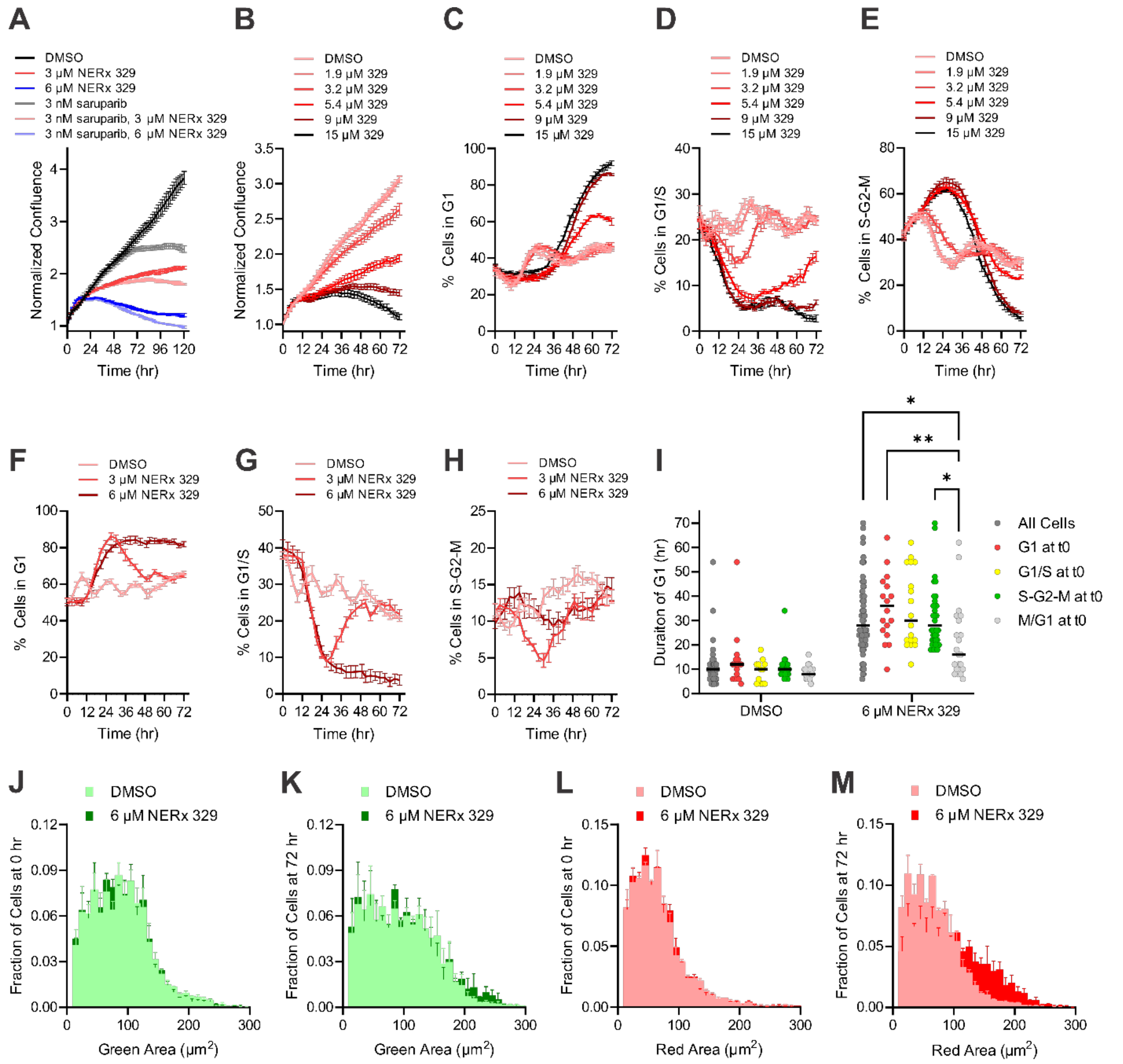

Figure S7. A) Confluence of MDA-MB-436 FUCCI cells treated with vehicle control, NERx 329, saruparib, or combination as monitored by Incucyte live cell imaging. **B)** Confluence of MDA-MB-436 FUCCI cells treated with vehicle control or increasing concentrations of NERx 329 as monitored by live cell imaging. Quantification of MDA-MB-436 FUCCI cells in **C)** G1, **D)** G1/S, or **E)** S-G2-M following treatment with vehicle control or increasing concentrations of NERx 329. Quantification of NSCLC A549 FUCCI cells in **F)** G1, **G)** G1/S, or **H)** S-G2-M following treatment with vehicle control or increasing concentrations of NERx 329. **I)** Quantification of MDA-MB-436 FUCCI cell G1 durations following treatment with vehicle control or NERx 329. Populations are divided according to the cell cycle phase that each cell was

in upon the initiation of treatment. Area of green fluorescence at **J)** the start of treatment and **K)** after 72 hours from MDA-MB-436 FUCCI cells treated with DMSO or NERx 329. Area of red fluorescence at **L)** the start of treatment and **M)** after 72 hours from MDA-MB-436 FUCCI cells treated with DMSO or NERx 329.
