# Supplementary material for: Replication protein A protects lagging strand gaps, restricting PARP inhibitor-induced synthetic lethality in BRCA1-deficient tumors
Source: Nucleic Acids Res. 2026 Apr 28;54(8):gkag396. doi: 10.1093/nar/gkag396 (PMC13122181; doi:10.1093/nar/gkag396)
Supplement: gkag396_Supplemental_Files [file gkag396_supplemental_files.zip › SuppMovieLegends.docx]

**Supplemental Movie Legends for:**

**Chemical inhibition of RPA gap protection sensitizes BRCA1-deficient cancers to PARP inhibition**

Pamela S. VanderVere-Carozza*, Matthew R Jordan*, Joy E. Garrett, Karen E. Pollok, Katherine S. Pawelczak and John J. Turchi

**Supplemental Movie Legends**

**Supplemental Movie 1.** Representative movie of MDA-MB-436 FUCCI cells treated with vehicle control. Images taken every 2 hours for 62 hours total.

**Supplemental Movie 2.** Representative movie of MDA-MB-436 FUCCI cells treated with NERx-329. Images taken every 2 hours for 62 hours total.

**Supplemental Movie 3.** Representative movie of MDA-MB-436 FUCCI cells going through mitotic bypass upon treatment with NERx-329. Cell undergoing mitotic bypass is indicated by the blue square. Images taken every 2 hours for 72 hours total.
